# Complications and management of forgotten long-term biliary stents

**DOI:** 10.3748/wjg.v23.i4.622

**Published:** 2017-01-28

**Authors:** Se Hoon Sohn, Jae Hyun Park, Kook Hyun Kim, Tae Nyeun Kim

**Affiliations:** Se Hoon Sohn, Jae Hyun Park, Kook Hyun Kim, Tae Nyeun Kim, Division of Gastroenterology and Hepatology, Department of Internal Medicine, Yeungnam University College of Medicine, Daegu 42415, South Korea

**Keywords:** Biliary stent, Long-term complications, Forgotten stents, Acute cholangitis, Biliary stone

## Abstract

**AIM:**

To evaluate complications and management outcomes of retained long-term plastic biliary stents.

**METHODS:**

Endoscopic plastic biliary stent placement was performed in 802 patients at Yeungnam University Hospital between January 2000 and December 2014. Follow-up loss with a subsequently forgotten stent for more than 12 mo occurred in 38 patients. We retrospectively examined the cause of biliary stent insertion, status of stents, complications associated with biliary stents and management outcomes of long-term plastic biliary stents. Continuous variables were analyzed using the *t* test. Observed frequencies in subsets of the study population were compared using Fisher’s exact test and χ^2^ tests. Statistical significance was defined as *P* < 0.05 (two-tailed).

**RESULTS:**

Mean age of patients was 73.7 ± 12 years and male-to-female ratio was 2.2:1. Indications of plastic biliary stent insertion were bile duct stones (63.2%, 24/38) and benign bile duct stricture (52.6%, 20/38). Mean duration of retained plastic stent was 22.6 ± 12.2 mo, and in 10 cases (26.3%), stents were retained for more than 24 mo. Common bile duct (CBD) stones or sludge were found in most cases (92.1%, 35/38). The most common complication was acute cholangitis (94.7%, 36/38). Stent removal by endoscopic approach was successfully performed in 92.1% (35/38) of the cases. In 3 cases, an additional plastic stent was inserted alongside the previous stent due to failure of the stent removal. Endoscopic removal of bile duct stones was successful in 73.7% (28/38) of the cases. When patients were divided into two groups by duration of stent placement (12 to 24 mo *vs* over 24 mo), there were no differences in the development of cholangitis, presence of biliary stones, and success rate of endoscopic removal of stones and biliary stents.

**CONCLUSION:**

The most common complication of retained long-term plastic biliary stents was acute cholangitis associated with CBD stones. Endoscopic management was successfully performed in most cases.

**Core tip:** There is little information on the consequence of retained long-term biliary stents and management of complications. In this study, follow-up loss with a subsequently forgotten stent for more than 12 mo occurred in 38 patients and cholangitis due to common bile duct stones or sludge developed in most cases (94.7%, 36/38). Stent removal by endoscopic approach was successfully performed in 92.1% (35/38) of the cases.

## INTRODUCTION

Endoscopic retrograde cholangiopancreatogram (ERCP) with endoscopic sphincterotomy (EST) and stone extraction is widely accepted as the treatment of choice for a patient of any age with choledocholithiasis. This technique has been reported to be successful in 80%-95% of the cases[1-3]. Endoscopic removal of biliary stones may infrequently be impossible despite improved ERCP techniques, especially when large or impacted stones are present, or in cases of a coexisting narrowing of the distal common bile duct (CBD). Surgical procedures could be options for patients who failed endoscopic restoration of bile drainage. Endoscopic insertion of biliary endoprosthesis has been proposed as an alternative for elderly patients or those with high surgical risks[4-7].

Biliary stents are tubular devices made of plastic or metal, and are used primarily to establish patency of an obstructed bile duct caused by malignancy, benign biliary strictures, or bile duct stones. Early complications of biliary stents are infection, pancreatitis, and bleeding; most late complications are stent dysfunction, and much less frequently cholecystitis, duodenal perforation, and bleeding[8]. The value of short-term biliary stenting has been proven, but the benefit of long-term stenting is less clear. Several studies have shown the effectiveness of long-term biliary endoprostheses in the management of irretrievable CBD stones in high-risk elderly patients[4-7]. In these studies, the long-term complications, such as occlusion, stent migration or cholangitis, increased with time and replacement or removal of the biliary stents was recommended after 3-6 mo[9]. In a study with 58 patients who underwent biliary endoprostheses as a permanent treatment, complications (most commonly cholangitis) occurred in 40% of patients, and 16% of patients died because of biliary-related causes[10]. In these studies, clinical symptoms and laboratory tests were followed-up regularly by physicians.

Although there are several case reports, little is known about what happens when biliary stents were forgotten or omitted by patients and consequently retained for a period of time greater than 12 mo. The aim of this study was to evaluate complications of retained long-term biliary stents and management outcomes in patients with a plastic biliary stent left unnoticed for longer than 12 mo.

## MATERIALS AND METHODS

Endoscopic plastic biliary stent placement was performed in 802 patients at Yeungnam University Hospital between January 2000 and December 2014. The indications of tubal catheterization were failure of stone removal and coexistence of benign bile duct narrowing. Among them, follow-up loss with a subsequently forgotten stent for more than 12 mo happened in 38 patients.

Patient characteristics, plastic biliary stent indication, status and complications of retained long-term biliary stents, and management outcomes were reviewed retrospectively. Patients were divided into two groups by stent placement duration: a 12-mo to 24-mo group, and an over 24-mo group. Various factors were compared between the two groups.

All patients with retained long-term plastic biliary stents were evaluated with ERCP followed by a computed tomography scan. ERCP was performed with a standard side-viewing duodenoscope (TJF-140; Olympus, Tokyo, Japan) by experienced endoscopists. After taking a cholangiogram, removal of biliary endoprosthesis was attempted using a stone retrieval basket. In cases of coexisting biliary stones, endoscopic stone removal was performed using conventional methods. When stent removal failed, an additional plastic biliary stent (Cotton-Leung biliary stents; Wilson-Cook Medical, Winston-Salem, NC, United States) was inserted alongside the previous stent to treat cholangitis. When complete stone removal failed or biliary stricture was found, a plastic biliary stent was inserted to prevent cholangitis. Complete stone removal was confirmed by cholangiogram at the end of each procedure.

All statistical analyses were performed using the Statistical Program for Social Sciences (SPSS 18.0 for Windows; SPSS Inc., Chicago, IL, United States). Continuous variables were analyzed using the *t* test. Observed frequencies in subsets of the study population were compared using Fisher’s exact test and χ^2^ tests. Statistical significance was defined as *P* < 0.05 (two-tailed).

## RESULTS

Clinical characteristics of patients are summarized in Table 1. Mean patient age was 73.7 ± 12.1 years (range: 38-92 years), and the male-to-female ratio was 2.2:1. Endoscopic retrograde biliary drainage (ERBD) was performed in 19 patients (50.0%) with CBD stones, 14 patients (36.8%) with bile duct strictures, and 5 patients (13.2%) with CBD stones combined with bile duct strictures. The mean duration of stent-stay was 22.6 ± 12.2 mo (range: 12-58 mo), and in 10 cases (26.3%) stents were in place for more than 24 mo. Most patients visited the hospital due to development of clinical symptoms (92.1%, 36/38), and 2 cases of retained biliary stents were found incidentally. Patients presented with abdominal pain (36/38), fever (20/38), jaundice (13/38), and abnormal liver function tests (13/38) (Table 2).

**Table 1:** Baseline patient characteristics *n* (%)

	**Total (*n* = 38)**
Age, mean, yr	73.7 ± 12.1
Sex, male/female	26(68.4)/12(31.6)
Indication of biliary stent	
CBD stone	19 (50.0)
CBD stricture	14 (36.8)
CBD stone with CBD stricture	5 (13.2)
Types of stents	
Diameter, Fr: 7/10/11.5	2(5.3)/21(55.3)/15(39.5)
Length, cm: 5/7/9	13(34.2)/22(57.9)/3(7.9)
Underlying diseases	
Chronic pancreatitis	4 (10.5)
Liver cirrhosis	1 (2.6)
STG BII	4 (10.5)
Chronic renal failure	2 (5.3)
Cerebrovascular accident	5 (13.2)

CBD: Common bile duct; STB BII: Subtotal gastrectomy with Billroth II.

**Table 2:** Presenting symptoms and laboratory findings at admission

**Variable**	**Total (*n* = 38)**
Symptoms	
Abdominal pain	36 (94.7)
Fever	20 (52.6)
Jaundice	13 (34.2)
Without symptom	2 (5.3)
Laboratory findings	
WBC, cells/mcL	10270.20 ± 4835.67
ALT, IU/L	122.50 ± 238.72
GGT, IU/L	236 ± 209.18
Total bilirubin, mg/dL	2.57 ± 2.19
Amylase, U/L	213.74 ± 494.75

Values are presented as mean ± SD or *n* (%). WBC: White blood cell; ALT: Alanine aminotransferase; GGT: Gamma-glutamyl transferase.

Complications and clinical outcomes of retained long-term biliary stents are described in Table 3. The most common complication was acute cholangitis (94.7%), followed by obstructive jaundice (34.2%), internal stent migration (13.2%), and pancreatitis (5.3%). In 36 patients with cholangitis, 35 cases were caused by CBD stones and 1 case by stent occlusion. Stent removal by endoscopic approach was performed successfully in 92.1% of the cases (35/38). All stent lumens were occluded with sludge and brown pigment stones, or sludge was adherent to the stent. An additional plastic stent was inserted alongside the previous stent in 3 patients with failed stent removal. Surgical management was considered for these patients, but was not performed due to comorbidities and generally poor patient conditions.

**Table 3:** Complications and outcomes according to biliary stent duration *n* (%)

**Variable**	**Total**	**1-2 yr**	**Over 2 yr**	***P* value**
	**(*n* = 38)**	**(*n* = 28)**	**(*n* = 10)**	
Age, yr	73.7 ± 12.1	75.6 ± 10.0	68.3 ± 16.1	0.104
Sex, male/female	26/14(68.4/31.6)	19/9(67.9/32.1)	7/3 (70/30)	1.000
Duration of stent-stay, mo	22.6 ± 12.2	16.4 ± 3.0	39.7 ± 11.7	0.001
Complications of stents				
Jaundice	13 (34.2)	11 (39.3)	2 (20.0)	0.441
Cholangitis	36 (94.7)	26 (92.9)	10 (100.0)	1.000
Pancreatitis	2 (5.3)	1 (3.6)	1 (10.0)	0.462
Internal migration	5 (13.2)	3 (10.7)	2 (20.0)	0.592
Presence of CBD stone	35 (92.1)	25 (89.3)	10 (100.0)	0.552
Endoscopic treatment				
Stone removal	28 (73.7)	21 (75.0)	7 (70.0)	1.000
Stent removal	35 (92.1)	26 (92.9)	9 (90.0)	1.000
Stent reinsertion	19 (50.0)	16 (57.1)	3 (15.8)	0.269
Additional stent insertion	3 (7.9)	2 (7.1)	1 (10.0)	1.000

OR: Odds ratio; CI: Confidence interval; CBD: Common bile duct.

**Figure 1:**
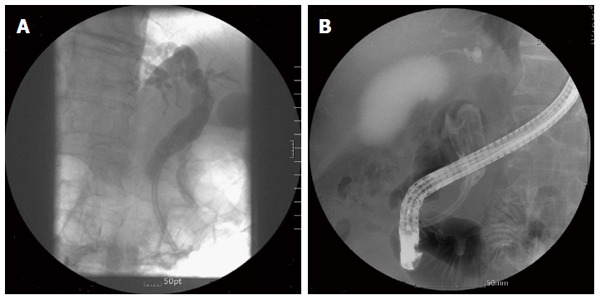
Stone formation 29 mo after stent insertion. A: Plastic biliary stent inserted for common bile duct stricture without stone; B: Large brown stone filled the bile duct.

**Figure 2:**
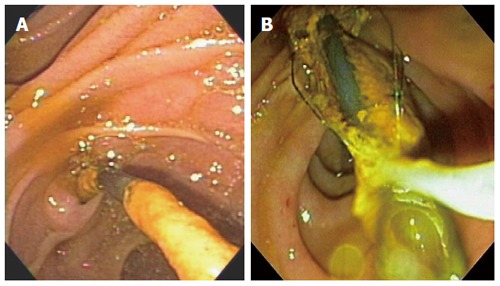
Brown stone stuck tightly to stent was removed by stone retrieval basket (A and B).

CBD stones were found in 35 patients (92.1%). Of the 14 patients who underwent ERBD due to CBD strictures and had no stones initially, CBD stones or sludge developed in 11 patients (78.6%) (Figure 1). Endoscopic CBD stone removal was successful in 73.7% of patients (28/38). Stone removal was performed by a stone retrieval basket and brown stones were stuck tightly to stents in most cases (Figure 2).

**Figure 3:**
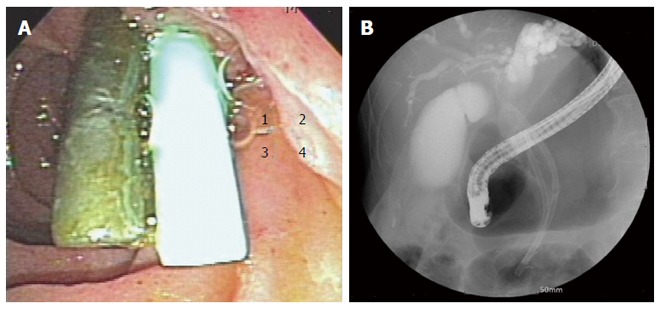
Additional stent inserted alongside previous stent (A) and large stones formed around the plastic biliary stent (B).

Retrieved CBD stones were brown pigment stones in all cases. In some patients, large stones formed around the plastic biliary stent and impacted at the CBD, which were impossible to remove endoscopically (Figure 3). For 10 patients with failed endoscopic stone removal or biliary strictures, biliary stents were reinserted to prevent cholangitis.

When patients were divided into two groups based on the duration of stent-stay, there were no significant differences between the 12-to-24-mo group and the over 24-mo group in development of cholangitis, pancreatitis, internal stent migration, CBD stone development, and overall success rates of endoscopic stone removal and biliary stent.

## DISCUSSION

The current standard treatment of bile duct stones is EST and stone extraction, followed by laparoscopic cholecystectomy if indicated. Surgical procedures such as bile duct exploration and sphincteroplasty, and drainage procedures such as choledochoduodenostomy and hepaticojejunostomy, are options for patients who fail endoscopic therapy[1-3].

Biliary endoprosthesis has been used for biliary drainage as a temporary measure or alternative for patients who are unfit for surgery. Biliary drainage is of great importance in maintaining bile flow in cases of stone impaction in the CBD. Placement of a biliary stent is essential for the treatment or prevention of acute cholangitis in this clinical setting, with a success rate of > 95%[1-3]. This procedure stabilizes the patient so that elective surgery or subsequent endoscopic treatment can be carried out when the patient’s status has improved, thus reducing procedural risks. In elderly patients or patients with severe comorbidities who are poor candidates for further endoscopic or surgical treatments, endobiliary stenting may serve as a permanent therapy; studies report that complication rates associated with surgery in elderly patients range from 12% to 28%[11-15]. In this study, poor surgical candidates who underwent endoscopic biliary stenting showed symptom relief and a low complication rate for more than 1 year, although most patients ultimately developed complications associated with the retained biliary stent itself.

Early outcomes of biliary stenting are well established and include good drainage and a low complication rate, but late outcomes remain uncertain. Most reports revealed that the success rate of endoscopic biliary stenting was nearly 100%, and that early morbidity was low and could be controlled well[16-18]. The major disadvantage of biliary stenting is clogging of the endoprosthesis. It is widely known that the mean patency duration of the plastic biliary stent is about 6 mo to 12 mo for benign diseases[19,20]. Replacement of plastic stents is therefore recommended every 3-6 mo to prevent cholangitis[8,9]. Biliary passage patency is maintained longer when stents are inserted for benign diseases such as biliary stone or benign biliary stricture, as compared to metal stents of malignancies. In this study, biliary stenting was performed for benign diseases, and the mean duration of stent placement was 22.55 ± 12.16 mo before developing symptoms or complications. The plastic biliary stent does not serve as the sole conduit for bile duct flow when used for benign diseases. As a passage between CBD and stent was retained in benign diseases, this passage may provide a conduit for bile flow even when the stent is completely obstructed[10,21,22]. Stent occlusion is the main cause of malignant biliary obstruction, and symptoms and complications occur earlier.

Although biliary patency may be maintained for a longer period after plastic biliary stent insertion, long-standing biliary stents consequentially increase the risk of cholangitis due to formation of biliary stones, because the biliary stent itself may serve as a nidus for stone formation. Lost sphincter of Oddi function, a mechanical barrier preventing regurgitation of duodenal contents into the bile duct, results in bacterial growth in the bile duct by ascending infection and plays an important role in the formation of brown pigment stones[23-25]. All stones in this study appeared to be brown pigment stones. Brown stones were distinguishable by their reddish brown to dark brown color and muddy appearance in macroscopic findings seen at endoscopy.

In this study, 92.1% of patients presented with symptoms of cholangitis, which most often occurred due to development of a CBD stone rather than stent occlusion. Of the 19 patients who underwent stenting for biliary stricture and had no stones initially, 16 patients (84.2%) developed a biliary stone or sludge. In this study, biliary stones were found stuck to biliary stents. Based on this information, the biliary stent may serve as a nidus for stone formation and development, and stent occlusion could develop because of biliary stasis.

Several previous studies showed that biliary patency restoration was successfully achieved in more than 95% of patients with stent obstruction[26]. The follow-up duration for development of complications and clinical symptoms was relatively short in most studies.

Little is known about the complications and management of long-term biliary stents retained more than 1 year. A recent study of 5 cases of long-standing forgotten biliary stents with 45.5 mo (range: 23-84 mo) of stent-stay reported that 4 of them required surgery for treatment[27]. Another recently published article documented that 2 of 3 patients (75%) underwent surgery for treatment of long-stayed forgotten stents[28-30]. In this study, however, 35 of 38 patients (92.1%) with retained long-term biliary stents were successfully treated by endoscopic approach. In 3 cases of endoscopic stent removal failure in patients who were unfit for surgery, cholangitis could be controlled by additional biliary stent placement without surgery.

In conclusion, the most common complication of retained long-term plastic biliary stents was acute cholangitis associated with CBD stones. Endoscopic management was successfully performed in most cases. Biliary patency was likely to be maintained more than 1 year. The rate of complications, such as cholangitis or stent impaction, might be increased as the stent was in place for a longer duration, and CBD stone development ultimately occurred in most cases of long-standing biliary stent. All patients should be informed of the possibility of complications related to retained long-term endoprosthesis placement, and stent change or definite treatment should be considered within 1 year of stent placement. Endoscopic management should be the primary option for the long-term biliary stent, especially in patients with comorbidities or who are unfit for surgery.

## COMMENTS

### Background

Biliary endoprosthesis has been used for biliary drainage as a temporary measure or alternative for patients who are unfit for surgery. Biliary drainage is of great importance in maintaining bile flow in cases of stone impaction in the biliary duct. In elderly patients or patients with severe comorbidities who are poor candidates for further endoscopic or surgical treatments, endobiliary stenting may serve as a permanent therapy.

### Research frontiers

There is little information on the consequence of retained long-term stayed forgotten biliary stents and management of complications, except a few case reports. In this study, the authors aimed to evaluate complications and management outcomes of retained long-term plastic biliary stents.

### Innovations and breakthroughs

The most common complication of retained long-term plastic biliary stents was acute cholangitis associated with common bile duct stones. Endoscopic management was successfully performed in most cases.

### Applications

In this study, long-term biliary stents were successfully treated by endoscopic approach. This study emphasizes the importance of patient follow-up and programmed withdrawal of stents. Endoscopic management could be the primary option for the long-term biliary stent, especially in patients with comorbidities or who are unfit for surgery.

### Terminology

Biliary stents are tubular devices made of plastic or metal, and are used primarily to establish patency of an obstructed bile duct caused by malignancy, benign biliary strictures, or bile duct stones.

### Peer-review

The authors performed a good retrospective study of a cohort of patients hospitalized for choledocholithiasis. The authors are interested in the management and the complications related to the presence of forgotten long-term biliary stents of more than 1 year. The authors collected a good amount of cases and the results can be useful information for the gastroenterologist.
